# Machine learning predicts weight change in a real-world digital health application

**DOI:** 10.1186/s12916-026-05320-x

**Published:** 2026-10-27

**Authors:** Christina Glasbrenner, Marco Lehmann, Christoph Höchsmann, Felix Schirmann, Karsten Koehler

**Affiliations:** 1 TUM School of Medicine and Health, Department of Health and Sport Sciences, Technical University of Munich, Munich, Germany; 2 Oviva AG, Berlin, Germany

**Keywords:** Digital health, Digital therapeutics, Mobile applications, Obesity medicine, Weight loss

## Abstract

**Background:**

Digital health applications offer scalable support for weight loss, but individual outcomes and retention vary substantially. It remains unclear how predictive information evolves during treatment, whether app engagement adds predictive information beyond early weight response, and whether the same factors predict weight loss and retention.

**Methods:**

Using real-world data from the 12-week digital health application *Oviva Direkt for Obesity*, we developed machine learning models to predict clinically meaningful weight loss, continuous relative weight change, and full app retention. Weight-change and full-retention analyses included 17,953 and 26,714 adults, respectively. Models evaluated baseline characteristics, 4-week weight, and weekly app engagement metrics separately and in combination to quantify their incremental predictive value and assess how performance changed when early-treatment information was added to pretreatment characteristics.

**Results:**

Baseline characteristics alone showed limited discrimination for clinically meaningful weight loss at weeks 8 and 12 (ROC AUC 0.54–0.58). Adding 4-week weight substantially improved performance (ROC AUC 0.86–0.92), whereas app engagement alone yielded modest improvements (ROC AUC 0.63–0.67). Adding app engagement to models including 4-week weight provided little further improvement (ROC AUC 0.87–0.92). A similar pattern was observed for continuous weight-change prediction. In contrast, full-retention prediction improved from ROC AUC 0.81 with baseline characteristics to 0.90 with app engagement, while 4-week weight provided no additional benefit. Weight-loss prediction was driven primarily by baseline weight, 4-week weight, and self-defined target weight, whereas engagement-related variables like goal setting and personalized logging recommendations were more influential for full retention.

**Conclusions:**

Predictive performance for weight-loss outcomes improved substantially when information from the first four weeks, particularly 4-week weight, was incorporated. Accordingly, the week 8 and week 12 models should be interpreted as predictions updated during treatment rather than predictions based solely on pretreatment characteristics. Weight-loss response and sustained intervention use were characterized by distinct predictive information and may require different forms of early, targeted support. These findings provide a basis for further research on the early identification of individuals at risk of suboptimal outcomes and the development of more personalized digital obesity care. External validation in independent, relevant cohorts is required before the models can be considered for clinical implementation.

**Supplementary information:**

The online version contains supplementary material available at https://doi.org/10.1186/s12916-026-05320-x.

## Background

Overweight and obesity have increased substantially over recent decades and are projected to affect nearly half of the global adult population by 2030 [1]. Excess weight is associated with an elevated risk of non-communicable diseases, including type 2 diabetes, cardiovascular disease, and several cancers [1, 2]. Importantly, even modest weight loss (3–5% of initial body weight) is associated with clinically meaningful improvements in health outcomes [3–5]. Although multiple strategies for weight loss exist, lifestyle interventions remain the first-line non-pharmacological standard of care [6–8]. However, treatment responses are highly heterogeneous, with substantial interindividual variability in both the magnitude and trajectory of weight change [9].

The mechanisms underlying this variability are multifactorial, reflecting complex interactions among demographic, metabolic, psychological, behavioral, environmental, and other factors [10, 11]. In addition, most predictive approaches rely on average treatment effects [12], limiting their ability to anticipate individual outcomes and tailor interventions accordingly [13].

Traditional statistical methods are limited in their ability to capture nonlinear relationships and complex interactions among predictors [14], underscoring the need for analytic approaches that can address this complexity and heterogeneity of weight change more effectively. Machine learning (ML) can capture nonlinear relationships and high-order interactions that traditional methods miss [15, 16]. We have previously shown that ML can accurately predict individual weight loss using comprehensive, resource-intensive measurements obtained under controlled conditions [17]. However, these prior approaches relied mainly on research-grade, highly detailed physiological and metabolic measurements (e.g., from controlled intervention studies such as CALERIE™ [17]), raising the question of whether comparable predictive performance can be achieved with routinely collected, real-world data, particularly app engagement metrics from scalable digital health applications.

Previous research has shown that early weight change is an important predictor of weight-loss success and that engagement with digital interventions is associated with treatment outcomes [18–20]. However, important questions remain regarding how routinely collected intervention data can best support individualized digital obesity care. In particular, the incremental predictive value of early intervention data beyond early weight change and whether information available early in treatment can predict subsequent weight change remain less well characterized. Moreover, relatively few studies have directly compared the predictors of weight-loss response and retention within the same real-world digital intervention [21, 22].

In this study, we applied ML to a large real-world dataset from the 12-week digital health application for weight management *Oviva Direkt for Obesity*, delivered via a mobile app, to predict individual weight-loss outcomes and full retention of app usage (i.e., sustained participant activity in the app during the final phase of the intervention). The efficacy of the intervention has previously been demonstrated in two randomized controlled trials [23, 24]. *Oviva Direkt for Obesity* is fully reimbursed within the German health care system as a digital health application and has been prescribed 455,000 times to date [25]. We aimed to address three complementary questions relevant to the use of routinely collected data for individualized digital obesity care: (1) how much additional predictive information becomes available during the first four weeks of the intervention compared with baseline information alone; (2) whether early intervention data provide predictive information beyond early weight change; and (3) whether the predictors of weight-loss response differ from those of full retention. To address these questions, predictions were based on baseline characteristics, early weight change, and early app engagement data. We examined three outcomes: clinically meaningful weight loss, continuous relative weight change, and full retention. We compared a range of widely used linear and tree-based ML models and applied interpretable ML approaches, including SHapley Additive exPlanations (SHAP), to identify the factors contributing most strongly to individual predictions.

Early identification of patients at increased risk of limited weight loss, disengagement, or both could enable more targeted and personalized support within digital behavior change interventions. For example, information available at the end of week 4 could help the treatment team identify patients at increased risk of not achieving clinically meaningful weight loss by week 12 or of disengaging during the remainder of the intervention, thereby enabling a more detailed assessment of individual needs and, where appropriate, providing tailored support.

## Methods

### General approach

This study aimed to identify early predictors of individual weight change and full retention within a scalable, 12-week digital health application for weight management. Specifically, we investigated how routinely collected baseline characteristics, early weight change, and app engagement metrics can be used to predict individual intervention outcomes and to characterize when and which types of information become most informative during the intervention. In particular, we examined (1) how predictive performance changes when early intervention data are added to baseline information, (2) whether early intervention data provide predictive information beyond early weight change, and (3) whether the predictors of weight-loss response differ from those of full retention. To capture complementary dimensions of intervention outcomes, we defined three prediction tasks: (a) binary classification of clinically meaningful weight loss of 3% and 5% at weeks 4, 8, and 12 (responder analysis); (b) regression of relative weight change at weeks 4, 8, and 12; and (c) binary classification of full retention, defined as sustained participant activity in the app during the final phase of the intervention (weeks 9–12).

The analysis was conducted using a large (*n* = 17,953) real-world dataset of patients across Germany who used *Oviva Direkt for Obesity*, a 12-week digital behavior change intervention for weight management, and consented to their data being used for research purposes. The selected dataset reflects real-world app usage and includes a large sample, providing a robust basis for model development and internal evaluation within the studied intervention setting. As this study involved the secondary use of anonymized data, ethical review was subject to a waiver by the ethical commission of the Technical University of Munich.

Our analytical framework followed a multi-step ML pipeline, including dataset preparation, feature and model selection, model training, and performance evaluation [17, 26]. To enhance the interpretability of our models, we quantified the contribution of each predictor using feature importance methods.

### Oviva direkt for obesity intervention

*Oviva Direkt for Obesity* is a self-guided, app-based, multimodal digital health application for weight management targeting diet, physical activity, and obesity-related psychological and behavioral factors. In the present study, the intervention was delivered over 12 weeks through a smartphone application.

The application comprised four main components: self-monitoring, self-management, education, and feedback. Self-monitoring functions enabled patients to record body weight, meals, physical activity, and other health-related parameters and to monitor change and progress over time. Self-management was supported through tasks and goals related to diet, physical activity, and other health behaviors, as well as a longer-term personal target weight. Educational content was provided weekly in text, audio, and video formats and covered nutrition, physical activity, and behavioral and psychological aspects of weight management, including stress management, emotional eating, and sleep. Feedback was provided based on self-monitoring data, completed tasks and goals, and progress over time, and included dietary suggestions, graphical progress displays, and weight trajectories.

These components incorporated behavior-change techniques, including goal setting, self-monitoring of behavior and outcomes, feedback on behavior and outcomes, and prompts or cues. For example, meal logging facilitated self-monitoring of dietary behavior, while graphical summaries and personalized feedback enabled patients to review their behavior and progress over time.

Patients independently progressed through the application content and completed self-monitoring and self-management activities without regularly scheduled therapeutic sessions. Professional support was available through a secure in-app chat: patients could contact a coach as needed, for example, when questions or difficulties arose, and coaches could also initiate contact proactively. Thus, the intervention was predominantly self-administered, with additional professional support available throughout the intervention.

A detailed description of the intervention components, their delivery modes, recommended uses, personalization, and corresponding variables analyzed in the present study is provided in Additional file 1: Table S1. Further details and efficacy evaluations of *Oviva Direkt for Obesity* have been published previously [23, 24].

### Problem definition

Binary classification models were developed to predict clinically meaningful weight loss (≥ 3% and ≥ 5%) at 4, 8, and 12 weeks [3]. The distribution of patients achieving these thresholds is presented in Additional file 1: Fig. S1. We used the same approach to predict full retention, defined as sustained participant activity in the app during the final phase of the intervention (weeks 9–12), as indicated by any recorded app engagement (e.g., weight logging, patient communication) in each of the last 4 weeks of treatment, which is an important outcome variable in digital health applications. In addition, regression models were trained to predict relative weight change from baseline at 4, 8, and 12 weeks (Additional file 1: Fig. S2).

Across all prediction tasks, model interpretability analyses were conducted to identify factors driving interindividual variability in outcomes.

### Dataset preparation

**Fig. 1:**
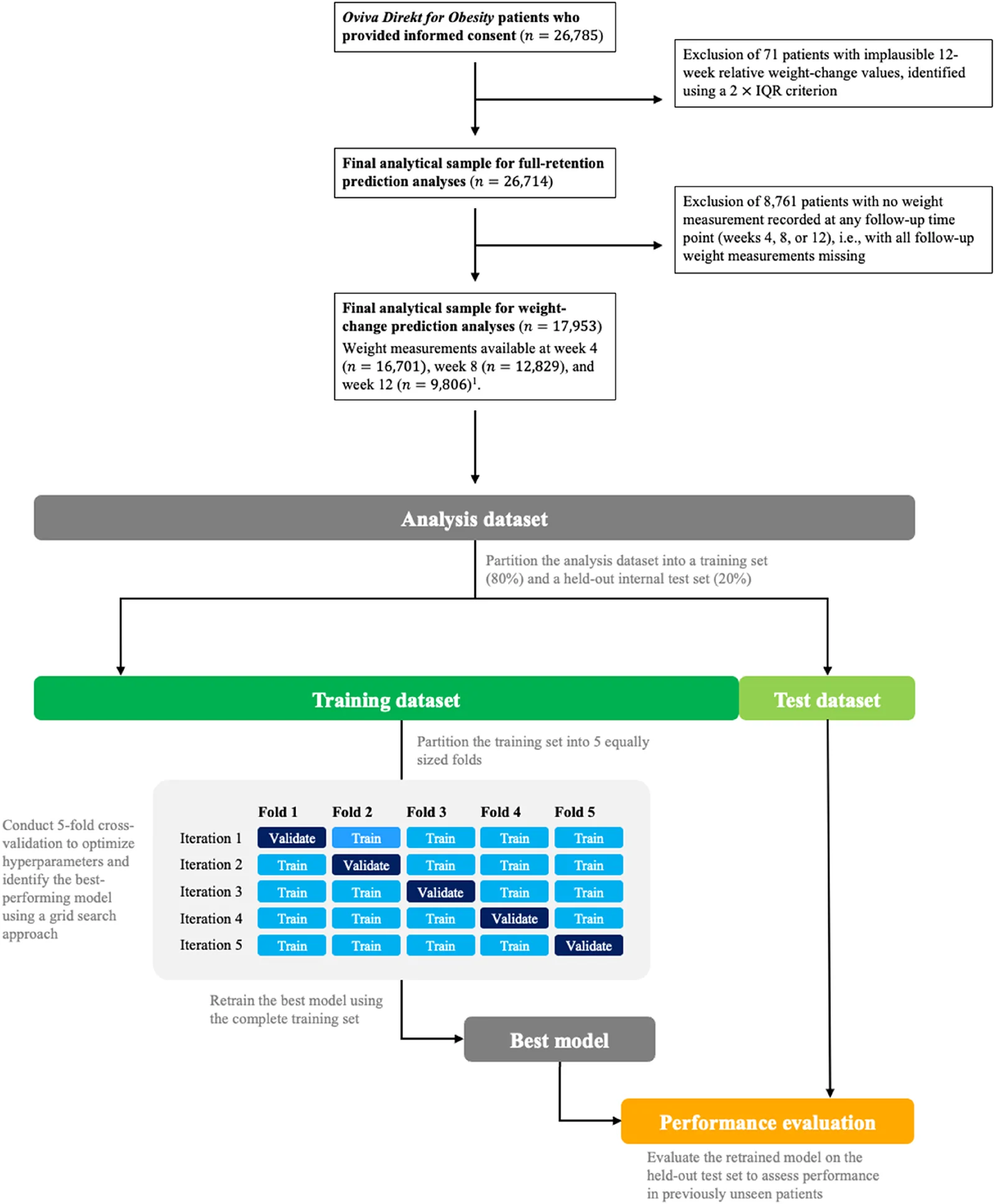
Participant flow and model development. Overview of participant inclusion and derivation of the analytical sample, dataset partitioning, model training and hyperparameter optimization, and final performance evaluation. ^1^The time-point-specific samples were not mutually exclusive; patients could contribute data to more than one sample. Abbreviation: IQR, interquartile range

Of the 26,785 patients using *Oviva Direkt for Obesity* who provided informed consent, 8,761 (33%) were excluded from the weight-change prediction analyses because no weight measurement was recorded at any of the three follow-up time points (weeks 4, 8, and 12), meaning that all three follow-up measurements were missing. An additional 71 patients were excluded because of implausible 12-week relative weight-change outcomes, defined as values exceeding twice the interquartile range. The final analytical sample included 17,953 patients. Of these, 16,701 had weight data reported at week 4, 12,829 at week 8, and 9,806 at week 12; these time-point-specific samples were not mutually exclusive (Fig. 1).

Baseline characteristics and selected app engagement metrics during weeks 1–4 were compared between patients included in and those excluded from the weight-change prediction analyses (Additional file 1: Table S2). The magnitude of between-group differences was quantified using absolute standardized mean differences (SMDs), with values below 0.1 commonly interpreted as indicating negligible differences [27].

For the full-retention analysis, all patients who provided informed consent, except the 71 patients with implausible weight data, were included (*n* = 26,714).

Age was collected through a self-report questionnaire provided during patient onboarding. Completion of this questionnaire was optional, and age was therefore not available for all patients. Age was missing for 69.7% of patients in the full-retention population and 57.2% in the overall weight-analysis population (Additional file 1: Table S3). To explore whether missing age data were associated with observed patient characteristics, engagement patterns, or the availability of follow-up weight measurements, patients with and without recorded age were compared using absolute SMDs (Additional file 1: Table S4).

Missing data for continuous variables were imputed using Multiple Imputation by Chained Equations (MICE) [28]. All predictors were standardized to improve comparability. To mitigate data leakage, both imputation and standardization were conducted exclusively during model training.

### Feature selection

For outcomes assessed at weeks 8 and 12, we evaluated four predictor sets to distinguish the predictive contribution of early weight information from that of app engagement: (1) baseline characteristics collected at the start of the intervention (sex, age, height, baseline weight, and self-defined target weight); (2) baseline characteristics plus 4-week weight; (3) baseline characteristics plus weekly app engagement metrics recorded during the first four weeks of the intervention, such as time spent on educational modules or the number of coach messages; and (4) baseline characteristics plus both 4-week weight and early app engagement metrics. For outcomes assessed at week 4, only baseline characteristics were used as predictors.

To further assess whether information available at week 4 could predict subsequent treatment response rather than primarily reflect weight loss that had already occurred, we conducted two additional secondary analyses. First, classification analyses were restricted to participants who had not yet achieved the respective 3% or 5% weight-loss threshold at week 4, and models predicted subsequent attainment of that threshold at weeks 8 and 12. Second, regression analyses predicted relative weight change occurring exclusively after week 4, from week 4 to week 8 and from week 4 to week 12. The same four predictor sets and modeling procedures were applied as in the primary analyses.

The engagement variables corresponded to the intervention components described in Additional file 1: Table S1 and included time spent engaging with educational content; patient-coach communication, including the number of coach messages, patient messages, and patient replies; personalized recommendations for the number of educational content items and frequencies for weight, meal, and physical activity logging; the number of goals set; and the number of reactions recorded in the app.

### Model selection

To ensure robust model selection, we evaluated a diverse set of widely used ML models, including both linear and tree-based approaches. For binary classification tasks, logistic regression, decision trees, random forests, and gradient boosting models were compared. Class imbalance was addressed using class-weighted loss functions. For regression analyses, linear models (including linear regression, Ridge, Lasso, and Elastic Net) and tree-based models (decision trees, random forests, and gradient boosting) were assessed.

### Model training

The data were randomly divided into a training set (80%) and a held-out test set (20%), with stratification applied to maintain class distributions. Within the training set, model development employed 5-fold cross-validation, in which the data were split into five equally sized folds; in each iteration, models were trained on four folds and validated on the remaining fold. This cross-validation approach was combined with a grid search to identify the best-performing model configuration (Fig. 1). Classification models were optimized using the area under the receiver operating characteristic curve (ROC AUC), and regression models using the root mean squared error (RMSE). The final model was then retrained on the full training dataset using the optimal hyperparameters.

### Performance evaluation

Model performance was assessed on the held-out test set, which was not used for model training or hyperparameter tuning. This evaluation represents internal holdout validation within the study population. For binary classification tasks, performance was evaluated using ROC AUC, accuracy, sensitivity, and specificity. For regression analyses, predictive performance was evaluated using bias, mean absolute error (MAE), and RMSE.

To assess whether the imputation of missing age values influenced model performance, we conducted sensitivity analyses in which the best-performing models from the primary analyses were refitted after excluding age as a predictor.

### Feature importance analysis

To evaluate the consistency of feature relevance across models, time points, and weight-loss thresholds, we performed a comparative feature-importance analysis based on feature rankings [29]. Model-specific importance measures were transformed into ordinal ranks (with rank 1 indicating the highest importance), and features were subsequently ordered by their mean rank across all evaluated scenarios.

To assess the stability of feature-importance rankings, we additionally evaluated the rankings of the top 10 features for the best-performing full-feature model across the five cross-validation folds. Mean rank and standard deviation, rank range, and the proportion of folds in which each feature ranked among the top 10 were summarized (Additional file 1: Table S5).

Feature contributions were further examined using SHAP values, which quantify the direction and magnitude of each feature’s contribution to class-specific predictions, with positive (negative) values indicating increased (decreased) model confidence for a given class [30].

### Implementation

Analyses were conducted in Python (version 3.12.6; Python Software Foundation, Beaverton, OR, USA). Machine learning algorithms were built with the *scikit-learn* library (version 1.5.2), and model interpretation was performed with the *shap* package (version 0.46.0) to assess feature-level impact on model predictions.

## Results

### Study population

Baseline characteristics of the study population are summarized in Table 1. The study cohort (*n* = 17,953) had a mean age of 53.8 ± 11.4 years (mean ± standard deviation) and was predominantly female (81%). Patients had a mean height of 169.0 ± 8.6 cm, a mean body weight of 100.0 ± 15.9 kg, and a mean body mass index (BMI) of 34.9 ± 4.2 kg/m^2^.

**Table 1:** Baseline characteristics of study participants

Characteristic	Total weight-analysis population (*n* = 17,953)	Weight-analysis subsample with available data at week 4 (*n* = 16,701)	Weight-analysis subsample with available data at week 8 (*n* = 12,829)	Weight-analysis subsample with available data at week 12 (*n* = 9,806)	Total full-retention population (*n* = 26,714)
Age (y)	53.8 (11.4)	53.9 (11.4)	54.3 (11.3)	54.6 (11.2)	53.5 (11.6)
Sex, n (%)					
Female	14,535 (81)	13,510 (81)	10,435 (81)	8,022 (82)	21,280 (80)
Male	3,418 (19)	3,191 (19)	2,394 (19)	1,784 (18)	5,434 (20)
Height (cm)	169.0 (8.6)	169.0 (8.6)	168.9 (8.5)	168.7 (8.4)	169.2 (8.7)
Weight (kg)	100.0 (15.9)	100.0 (15.9)	99.9 (15.8)	99.9 (15.7)	100.4 (16.2)
Body mass index (kg/m^2^)	34.9 (4.2)	34.9 (4.2)	35.0 (4.1)	35.0 (4.2)	35.0 (4.3)

Values are mean (SD) for continuous and n (%) for categorical variables

Compared with patients included in the weight-change prediction analyses, excluded patients were younger (47.7 vs. 53.8 years; absolute SMD = 0.496) and had substantially lower recorded app activity during weeks 1–4, including fewer patient messages, coach messages, and goals set (absolute SMDs ranging from 0.612 to 1.505). Differences in sex, height, baseline weight, baseline BMI, and self-defined target weight were small, with absolute SMDs below 0.1 (Additional file 1: Table S2).

Patients with and without recorded age showed relatively small differences in most baseline characteristics (absolute SMDs, 0.031–0.115), whereas larger differences were observed for selected early app engagement metrics (absolute SMDs, 0.326–0.570) and particularly for the availability of weight measurements at weeks 8 and 12 (absolute SMDs, 0.764 and 1.160, respectively; Additional file 1: Table S4).

### Predicting clinically meaningful weight loss

**Fig. 2:**
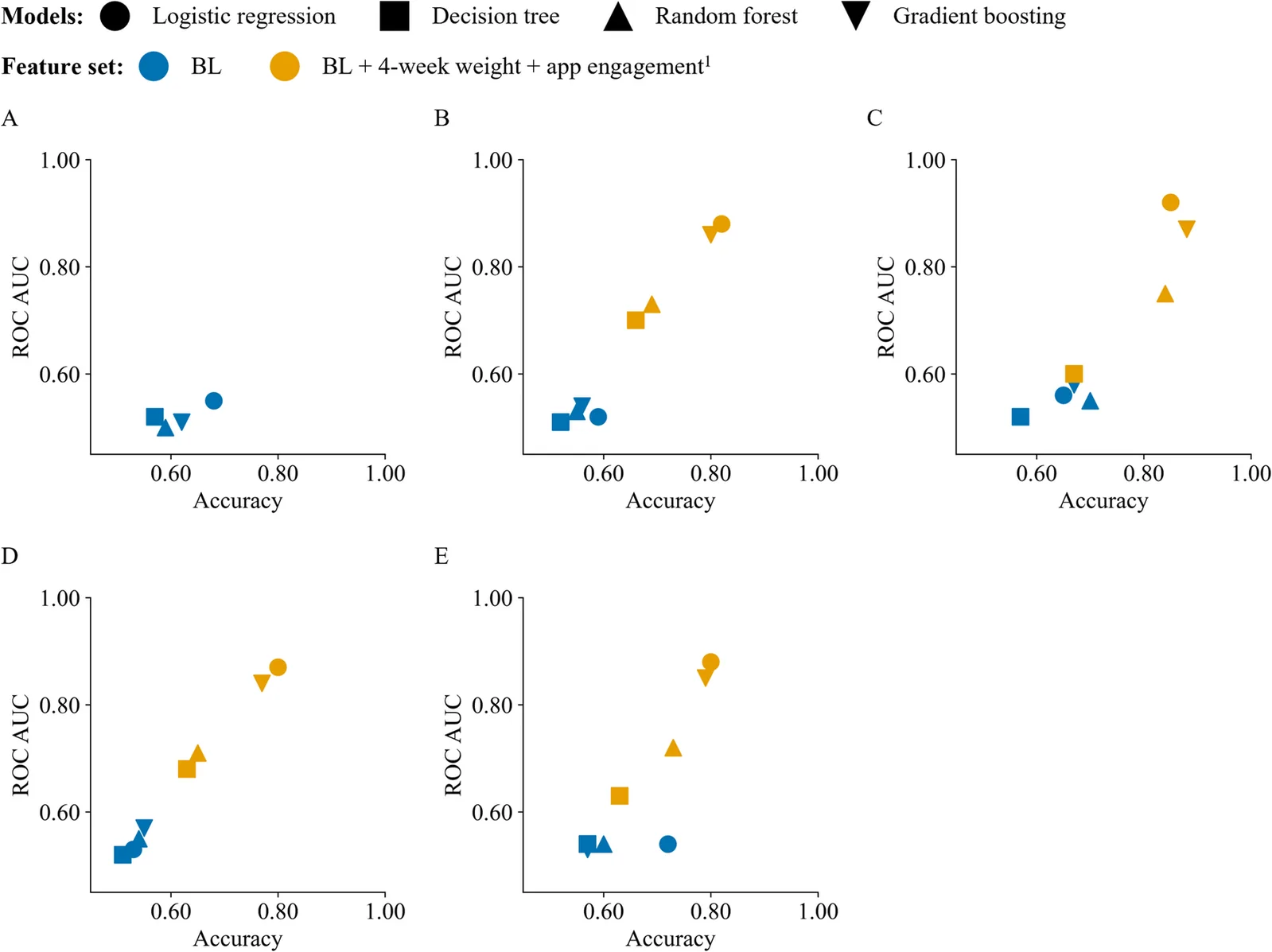
Binary classification performance for weight change prediction. Accuracy and ROC AUC for prediction of weight change at (**A**) 4 weeks (3% weight-loss threshold), (**B**) 8 weeks (3% threshold), (**C**) 8 weeks (5% threshold), (**D**) 12 weeks (3% threshold), and (**E**) 12 weeks (5% threshold). For outcomes at weeks 8 and 12, performance is shown for models using baseline variables only and models using baseline variables combined with 4-week weight and app engagement data from the first four weeks of the intervention. ^1^Weekly app engagement metrics recorded during the first four weeks of the intervention were included. Abbreviations: BL, baseline; ROC AUC, area under the receiver operating characteristic curve

Binary classification models based on baseline characteristics alone showed limited discrimination for predicting clinically meaningful weight loss at weeks 8 and 12, with ROC AUC values ranging from 0.54 to 0.58. The structured feature-set comparison showed that adding 4-week weight substantially improved discrimination, increasing ROC AUC to 0.86–0.92, whereas adding app engagement without 4-week weight resulted in more modest improvements (ROC AUC 0.63–0.67; Table 2). Combining 4-week weight and app engagement yielded ROC AUC values of 0.87–0.92. Thus, early weight information accounted for most of the improvement in predictive performance for subsequent clinically meaningful weight loss, while app engagement provided little additional discrimination once 4-week weight was available. Accuracy ranged from 0.55 to 0.68 for baseline-only models and from 0.80 to 0.85 for the full-feature models (Fig. 2). Detailed performance metrics for the baseline-only and full-feature models are provided in Additional file 1: Fig. S3.

**Table 2:** Predictive performance across feature sets for weight change and full retention prediction

Prediction outcome	BL	BL + 4-week weight		BL + app engagement^1^		BL + 4-week weight + app engagement^1^	
Clinically meaningful weight loss^2^, ROC AUC							
Week 8 (3% threshold)	0.54		0.88		0.66		0.88
Week 8 (5% threshold)	0.58		0.92		0.63		0.92
Week 12 (3% threshold)	0.57		0.86		0.67		0.87
Week 12 (5% threshold)	0.54		0.87		0.65		0.88
Relative weight change^2^, RMSE							
Week 8	0.0290		0.0228		0.0281		0.0223
Week 12	0.0315		0.0228		0.0302		0.0222
Full retention^2^, ROC AUC							
Weeks 9–12	0.81		0.81		0.90		0.90

^1^Weekly app engagement metrics recorded during the first four weeks of the intervention were included

^2^Only the performance of the best-performing model is shown for each prediction outcome and feature set. Higher ROC AUC values indicate better classification performance, whereas lower RMSE values indicate better regression performance

Abbreviations: BL, baseline; RMSE, root mean squared error; ROC AUC, area under the receiver operating characteristic curve.

To assess whether information available at week 4 could predict subsequent clinically meaningful weight loss rather than primarily identify participants who had already achieved the respective weight-loss threshold, we conducted additional classification analyses restricted to participants who had not yet achieved the respective 3% or 5% threshold at week 4. In these analyses, adding 4-week weight to baseline characteristics substantially improved discrimination for subsequent threshold attainment at weeks 8 and 12, increasing ROC AUC from 0.55 to 0.57 to 0.83–0.94. Adding app engagement without 4-week weight resulted in more modest discrimination (ROC AUC 0.61–0.65), while app engagement provided no further improvement once 4-week weight was included (ROC AUC 0.83–0.94; Additional file 1: Table S6).

Sensitivity analyses excluding age as a predictor showed largely unchanged classification performance, with absolute differences in ROC AUC ranging from 0.00 to 0.04 across prediction scenarios. For models incorporating the full feature set, differences were only 0.00-0.01 (Additional file 1: Table S7).

**Fig. 3:**
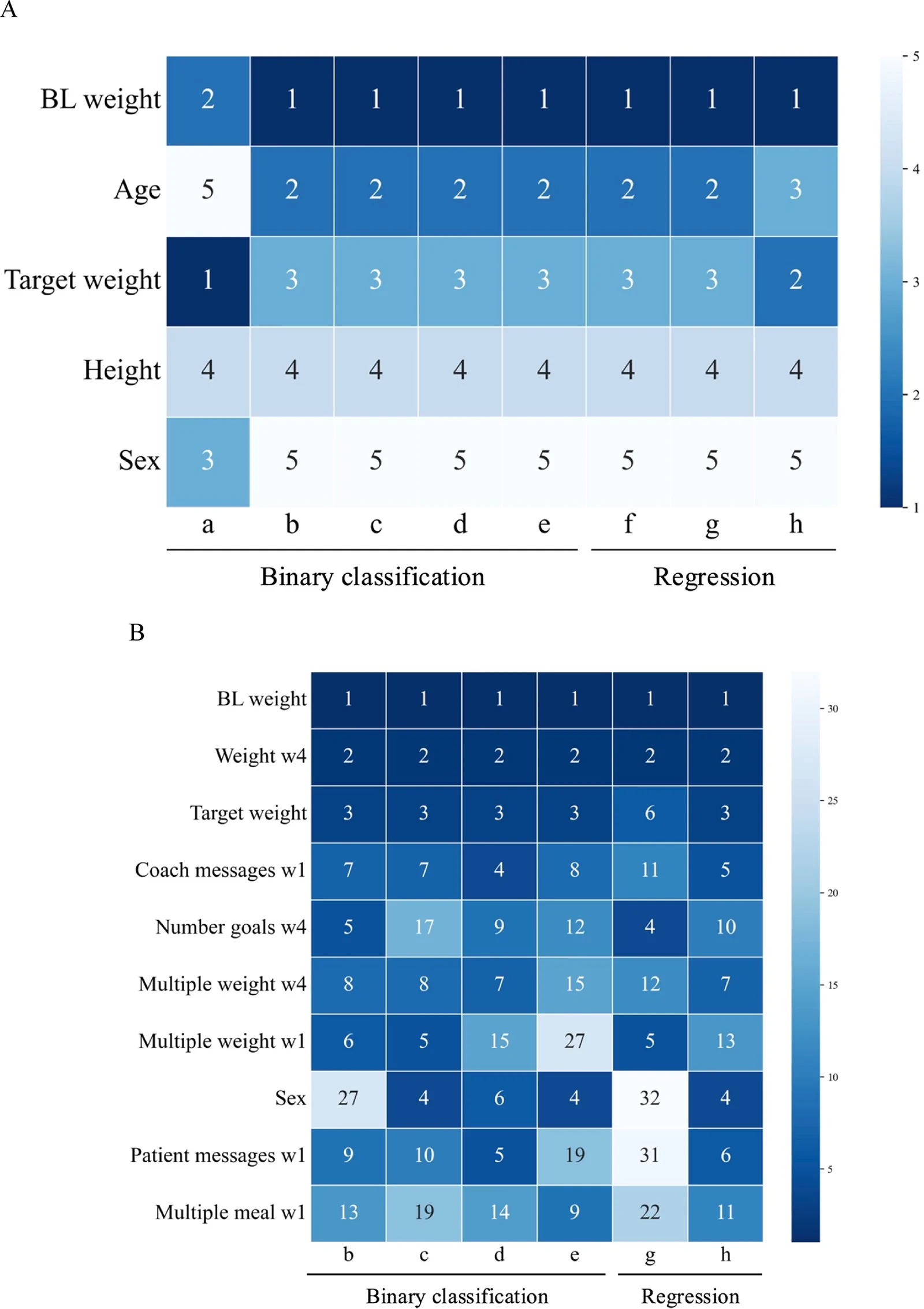
Feature importance across weight change prediction tasks. Heatmap showing feature importance rankings (1 = highest) for the best-performing model in each scenario (classification: highest ROC AUC; regression: lowest RMSE). Panels show feature rankings for models using (**A**) baseline variables only and (**B**) baseline variables combined with 4-week weight and app engagement data from the first four weeks of the intervention: (a) binary classification at 4 weeks (3% weight-loss threshold), (b) binary classification at 8 weeks (3% threshold), (c) binary classification at 8 weeks (5% threshold), (d) binary classification at 12 weeks (3% threshold), (e) binary classification at 12 weeks (5% threshold), (f) regression at 4 weeks, (g) regression at 8 weeks, and (h) regression at 12 weeks. Only features with a mean rank of ≤ 10 across scenarios are displayed. Scenarios (a) and (f) are not displayed in panel B because early intervention data are not available for prediction at week 4. Abbreviations: BL, baseline; RMSE, root mean squared error; ROC AUC, area under the receiver operating characteristic curve; w1, week 1; w2, week 2; w3, week 3; w4, week 4

Figure 3 summarizes the most influential features of the best-performing model in each scenario. In models restricted to baseline variables, baseline weight, age, and self-defined target weight emerged as the strongest predictors. When the full feature set was included, baseline weight and self-defined target weight remained among the most influential predictors, with 4-week weight additionally ranking among the top three predictors. Other top-ranked features (not shown separately for the classification) included the number of coach messages, personalized recommendations for the number of weight logs and meal logs, sex, the number of goals set, and the number of patient messages.

A more detailed examination of feature contributions was conducted using SHAP summary plots for models including all variables for predictions at 8 and 12 weeks (Additional file 1: Fig. S4). Baseline weight and 4-week weight were by far the most influential predictors. Higher baseline weight was associated with a greater likelihood of achieving the weight-loss threshold, whereas lower baseline weight was associated with a lower likelihood. Similarly, lower 4-week weight was associated with a higher probability of reaching the threshold, while higher 4-week weight indicated a lower probability. A lower self-defined target weight (i.e., a more ambitious weight-loss goal) was also associated with a higher likelihood of reaching the weight-loss threshold.

App engagement variables showed nuanced temporal patterns. A higher number of coach messages in week 1 and a higher number of goals set in week 4 were both associated with an increased likelihood of achieving the threshold. Notably, a higher number of patient messages in weeks 1 and 3 was associated with a lower likelihood of reaching the threshold, whereas more messages in week 4 were associated with a higher likelihood. A similar pattern was observed for personalized recommendations on the number of weight logs: higher numbers in weeks 1 and 2 were associated with a lower likelihood of reaching the threshold, while higher numbers in week 4 were associated with an increased likelihood.

Although several app engagement variables contributed to individual predictions in the full-feature models, the feature-set comparison indicated that their incremental contribution to overall discrimination was small once 4-week weight was available (Table 2).

### Predicting relative weight change

A similar pattern was observed for the prediction of continuous relative weight change. At weeks 8 and 12, adding 4-week weight to baseline characteristics reduced RMSE from 0.0290 to 0.0228 and from 0.0315 to 0.0228, respectively (Table 2). In contrast, adding app engagement without 4-week weight resulted in smaller reductions in RMSE, to 0.0281 at week 8 and 0.0302 at week 12. Combining 4-week weight and app engagement further reduced RMSE only slightly, to 0.0223 and 0.0222 at weeks 8 and 12, respectively. Consistent with the classification results, early weight information therefore accounted for most of the improvement in prediction of subsequent relative weight change, whereas app engagement provided limited additional predictive information once 4-week weight was available. Detailed model performance for the baseline-only and full-feature models is presented in Additional file 1: Fig. S5.

To further assess prediction of weight change that had not yet occurred, we additionally modeled relative weight change occurring exclusively after week 4. For subsequent weight change from week 4 to week 8, adding 4-week weight to baseline characteristics reduced RMSE from 0.0150 to 0.0143, whereas adding app engagement without 4-week weight resulted in an RMSE of 0.0147. The full feature set yielded an RMSE of 0.0142. For subsequent weight change from week 4 to week 12, adding 4-week weight reduced RMSE from 0.0226 to 0.0210, whereas app engagement without 4-week weight yielded an RMSE of 0.0227 and the full feature set an RMSE of 0.0212 (Additional file 1: Table S8). Thus, 4-week weight provided a modest improvement in predicting the magnitude of subsequent weight change, while app engagement provided little additional predictive information.

Regression performance was almost unchanged after excluding age as a predictor, with absolute differences in RMSE ranging from 0.000 to 0.001 across prediction scenarios (Additional file 1: Table S9).

Consistent with the binary classification analysis, baseline weight, age, and self-defined target weight were the most influential predictors in models restricted to baseline characteristics. When the full feature set was included, baseline weight, 4-week weight, and self-defined target weight were the three highest-ranked predictors, consistent with the strong predictive contribution of early weight information observed in the feature-set comparison. Additional influential predictors in the regression models (not shown separately) were very similar to those observed for classification, including the number of goals set in week 4, the number of coach messages in weeks 1 and 3, personalized recommendations for the number of weight (weeks 1 and 4) and meal logs (week 1), and learning time in week 3.

### Predicting full retention

**Fig. 4:**
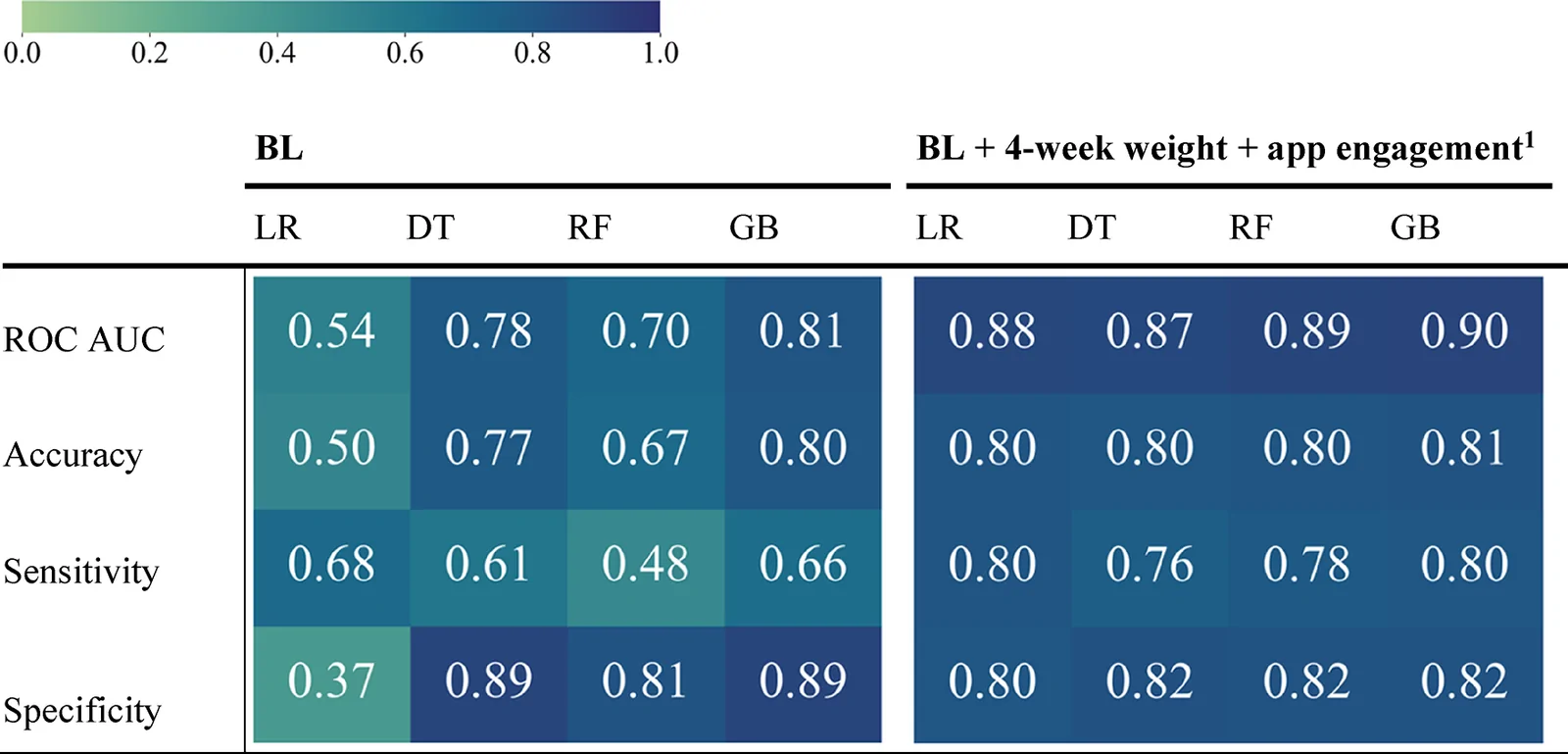
Performance metrics for full-retention prediction. Heatmap summarizing binary classification performance metrics for predicting full retention using baseline variables only and baseline variables combined with 4-week weight and app engagement data from the first four weeks of the intervention. ^1^Weekly app engagement metrics recorded during the first four weeks of the intervention were included. Abbreviations: BL, baseline; LR, logistic regression; DT, decision tree; RF, random forest; GB, gradient boosting; ROC AUC, area under the receiver operating characteristic curve

Full-retention prediction, defined as sustained participant activity in the app during the final phase of the intervention (weeks 9–12), achieved good discrimination using baseline characteristics alone (ROC AUC 0.81; Table 2). Adding 4-week weight did not improve performance (ROC AUC 0.81), whereas adding early app engagement to baseline characteristics increased ROC AUC to 0.90. Combining app engagement with 4-week weight did not provide further improvement (ROC AUC 0.90). Thus, in contrast to weight-loss prediction, the improvement in full-retention prediction was primarily attributable to early app engagement rather than early weight information. Detailed performance metrics for the baseline-only and full-feature models are shown in Fig. 4.

Sensitivity analyses excluding age as a predictor showed that the ROC AUC of the baseline-only model decreased from 0.81 to 0.64, indicating that age contributed substantial predictive information in this scenario. In contrast, performance remained similar for the model using the full feature set (ROC AUC, 0.90 vs. 0.89; Additional file 1: Table S10).

**Fig. 5:**
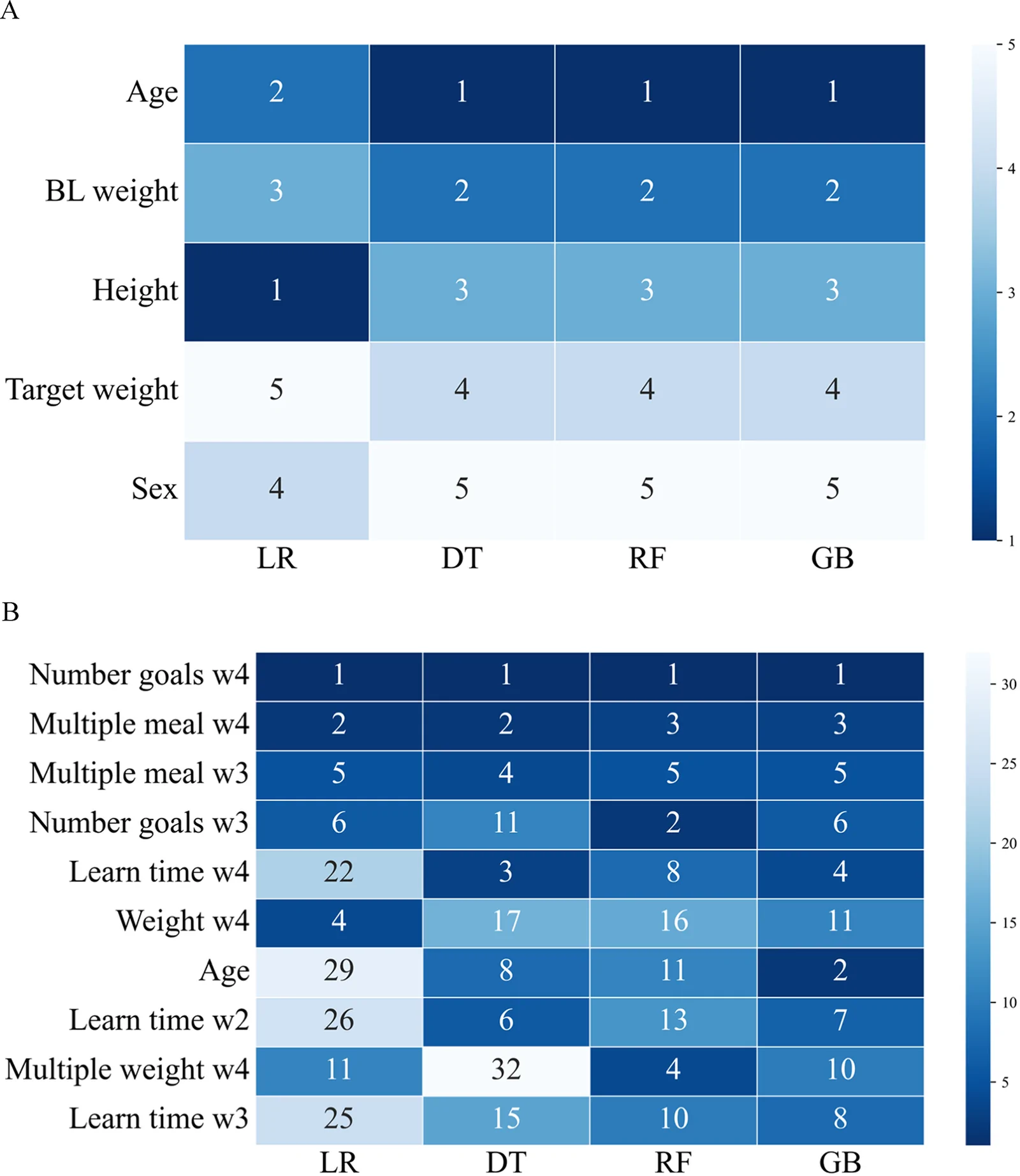
Feature importance across models predicting full retention. Heatmap showing feature importance rankings (1 = highest) across models predicting full retention. Panels show feature rankings for models using (**A**) baseline variables only and (**B**) baseline variables combined with 4-week weight and app engagement data from the first four weeks of the intervention. Only features with a mean rank of ≤ 10 across models are displayed. Abbreviations: BL, baseline; w1, week 1; w2, week 2; w3, week 3; w4, week 4; LR, logistic regression; DT, decision tree; RF, random forest; GB, gradient boosting

Figure 5 summarizes the most influential features for each model. In models restricted to baseline variables, age, baseline weight, and height emerged as the strongest predictors. When the full feature set was included, engagement-related features, including the number of goals set, learning time during the initial phase of the intervention, and personalized recommendations on the number of weight logs, emerged as the most important predictors. Notably, this predictor profile differed from that observed for weight-loss outcomes, for which baseline weight, 4-week weight, and self-defined target weight were most influential. Together with the feature-set comparison, these findings indicate that weight-loss response and full retention are characterized by different types of predictive information.

## Discussion

This study provides three main findings regarding the prediction of outcomes during a real-world digital health application for weight management. First, baseline characteristics alone provided limited predictive information for subsequent weight-loss response, whereas information available during the first four weeks substantially improved prediction. Second, most of this improvement was attributable to early weight information, while app engagement provided only limited additional predictive information for weight-loss outcomes once 4-week weight was available. Third, a different pattern emerged for full retention as app engagement substantially improved prediction, whereas 4-week weight provided little additional information. Consistent with this finding, the predictors of weight-loss response differed from those of full retention, with baseline weight, 4-week weight, and self-defined target weight being most influential for weight-loss outcomes and engagement-related variables playing a more prominent role in full-retention prediction. Together, these findings suggest that both the timing and type of information available during a digital health application are relevant for identifying patients at risk of different types of suboptimal outcomes.

The strong predictive contribution of early weight information is consistent with previous studies demonstrating that early weight change is an important predictor of subsequent weight-loss outcomes [18, 31–33]. In the present study, predictive performance improved substantially once 4-week weight became available compared with prediction based on routinely collected baseline characteristics alone in a large real-world digital intervention. Most of the improvement in weight-loss prediction from the baseline-only to the full-feature models at weeks 8 and 12 was attributable to the predictive information contained in 4-week weight. App engagement provided some additional information when added to baseline characteristics alone, but only limited incremental predictive value once 4-week weight was available. Accordingly, the models for weight-loss outcomes at weeks 8 and 12 should be interpreted as predictions updated during treatment that incorporate information from the first four intervention weeks, rather than as predictions based solely on pretreatment characteristics.

Feature-importance and SHAP analyses further supported the importance of early weight information, with baseline weight and 4-week weight among the strongest predictors of subsequent weight-loss outcomes. Baseline weight has previously been associated with long-term weight loss [18, 34], while greater initial weight loss has consistently been linked to greater subsequent weight loss [31, 32]. Self-defined target weight was also among the most influential predictors, consistent with previous evidence suggesting that more ambitious weight-loss goals may be associated with greater weight reduction [35, 36].

Importantly, the additional analyses indicated that 4-week weight did not merely identify participants who had already achieved the subsequent weight-loss outcome. When participants who had already reached the respective 3% or 5% weight-loss threshold at week 4 were excluded, information available on 4-week weight continued to provide substantial discrimination for subsequent threshold attainment at weeks 8 and 12 (ROC AUC, 0.83–0.94). Early weight information therefore also contained predictive information regarding future attainment of clinically meaningful weight-loss thresholds. In contrast, its contribution to predicting the exact magnitude of weight change occurring after week 4 was more modest. Together, these findings distinguish the strong ability of early weight response to identify participants likely to subsequently achieve clinically meaningful weight loss from the more difficult task of precisely predicting additional future weight change.

Although previous research has demonstrated associations between early app engagement and weight loss [37], our findings suggest that much of the predictive information for subsequent weight-loss outcomes available by week 4 is captured by early weight response itself. Nevertheless, several engagement variables contributed to individual predictions within the full-feature models. Their limited incremental contribution to overall weight-loss prediction should therefore be distinguished from their contribution to individual predictions and their potential relevance for understanding patterns of intervention use. Notably, a different pattern emerged for full-retention prediction, for which app engagement substantially improved model performance whereas 4-week weight provided little additional information. These findings suggest that early weight response and app engagement provide distinct types of predictive information depending on the intervention outcome being considered.

The SHAP analyses further indicated that the associations between individual engagement measures and subsequent weight loss varied across the early intervention period. During weeks 1–3, higher numbers of patient messages and personalized recommendations regarding weight logging were associated with a lower likelihood of achieving the weight-loss threshold. These signals may partly reflect emerging difficulties, uncertainty, or a greater need for support, but may also represent genuine engagement with the intervention. In contrast, higher values for selected engagement measures during week 4, including patient messages and personalized recommendations, were associated with an increased likelihood of achieving the weight-loss threshold. A higher number of goals set in week 4 and a higher number of coach messages in week 1 were also associated with an increased likelihood of achieving the threshold. These findings suggest that the context and timing of engagement may be relevant. However, engagement metrics are not inherently indicative of either difficulties or adherence and may reflect different underlying behaviors or support needs across patients and time points. The observed associations are correlational and should not be interpreted as evidence of specific behavioral mechanisms or causal relationships. These interpretations therefore require prospective validation.

A distinct pattern emerged for full retention. Prediction based on baseline characteristics alone achieved an ROC AUC of 0.81, and adding 4-week weight did not improve performance (ROC AUC 0.81). In contrast, adding early app engagement to baseline characteristics increased ROC AUC to 0.90, with no further improvement when 4-week weight was additionally included. Because both the early engagement predictors and the full-retention outcome are based on app-use behavior measured at different stages of the intervention, part of the observed predictive performance may reflect temporal persistence in individual engagement patterns. The strong discrimination should therefore be interpreted as showing that early patterns of app use are informative for subsequent sustained engagement, rather than as evidence that the identified engagement variables have causal effects on retention. One possible explanation for the stronger performance compared with the prediction of clinically meaningful weight loss using baseline features alone might be that the classes for full retention versus non-retention are more distinctly separable than those defined by weight-loss thresholds (e.g., ≥ 3% vs. <3%). In addition, the larger sample size used for this prediction (*n* = 26,714) may have enabled more robust learning of feature-outcome relationships compared with models predicting clinically meaningful weight loss (*n* = 17,953). Feature-importance analyses were consistent with these results. While baseline weight, 4-week weight, and self-defined target weight were particularly influential for weight-loss prediction, engagement-related variables, including the number of goals set and learning time during the initial phase of the intervention, were more prominent for full-retention prediction. Thus, weight-loss response and sustained intervention use appear to represent related but distinct dimensions of intervention success that are characterized by different types of predictive information.

A potential future use of the developed models would be to support risk stratification at the end of the first four intervention weeks. At this point, the treatment team could use information on early weight response and app engagement to identify patients at increased risk of not achieving clinically meaningful weight loss by week 12, disengaging during the remainder of the intervention, or both, while sufficient intervention time remains to consider additional support. However, whether such prediction-guided adaptations of digital obesity care improve treatment outcomes requires prospective evaluation and is beyond the scope of the present study [38]. Importantly, the type of support required may differ depending on the predicted outcome, given the distinct predictive patterns observed for weight-loss response and full retention. Patients at risk of disengagement may require support primarily aimed at sustaining participation, whereas those who remain engaged but show limited weight loss may require more intensive or individualized treatment support. This distinction may not be adequately addressed by “one-size-fits-all” strategies that apply the same type of support to all patients. For patients identified as being at risk of disengagement based particularly on the number of goals set, time spent engaging with educational content, and personalized recommendations regarding the number of weight logs during the initial phase of the intervention, engagement-focused support could include proactive outreach by a coach, early assessment of motivational, technical, or practical barriers to continued participation, and additional onboarding assistance. Reducing the number of required tasks, tailoring the frequency or format of educational content, and using strategically timed push notifications to encourage re-engagement may also help make participation more manageable [39, 40]. By contrast, patients who remain engaged but show limited weight loss may require support focused more directly on treatment response. This could include reviewing dietary intake and adherence, identifying barriers to implementing the intervention recommendations, refining behavioral goals, and providing more individualized dietary guidance or more intensive support from a coach or dietitian [41]. Where clinically appropriate, potential medical, psychological, medication-related, or social factors that may hinder weight loss could also be assessed. Patients showing both low engagement and limited weight loss may require a combination of engagement- and outcome-focused strategies. However, the identified predictors represent predictive associations and should not be interpreted as causal determinants. Increasing an individual engagement metric, such as the number of goals set or learning time, would therefore not necessarily improve retention or weight loss [42]. If externally validated, the models could subsequently be used to identify patients who may require additional support and to adapt the type of support provided according to whether the predominant concern is disengagement, limited weight loss, or both. For example, patients with a high predicted risk of disengagement could be prioritized for engagement-focused outreach, whereas patients with a high predicted risk of limited weight loss despite continued participation could be offered more intensive or individualized treatment support. Such a risk-stratified approach could help allocate intervention resources more efficiently and provide support at an earlier stage of treatment. Nevertheless, the proposed intervention strategies require prospective evaluation before they can be implemented in routine care.

Several limitations should be considered when interpreting these findings. First, the analyses were restricted to patients who consented to the use of their data for research purposes, representing only a subset of the available real-world data, which may have impacted representativeness. However, integrating available real-world data alongside evidence from randomized controlled trials is essential for a more comprehensive understanding of individual outcomes in weight-management interventions. Second, the study sample was predominantly female (81%) and comprised only patients in Germany, which may limit the generalizability of the results to other populations and settings. However, the large female share reflects the typical composition observed in many weight-management programs. Third, the developed models have not yet been externally validated. Although model performance was evaluated on a held-out test set that was not used during model development, this represents internal evaluation within the same underlying population and does not establish transportability to independent populations, healthcare settings, or other versions of the intervention. Consequently, the reported performance estimates may be optimistic relative to the performance that would be observed in independent external populations or settings [43]. External validation in an independent cohort representing a population and intervention setting relevant to the intended use of the models, with compatible definitions of the required predictors and outcomes, is therefore needed before broader clinical implementation can be considered. Fourth, age was collected through an optional self-report questionnaire during patient onboarding and was missing for 57.2% of patients in the overall weight-analysis population and 69.7% of patients in the full-retention population (Additional file 1: Table S3) and was therefore imputed, which may have influenced model performance and feature importance rankings, given that age is typically a strong predictor of weight loss [44, 45]. Comparisons between patients with and without recorded age showed relatively small differences in most baseline characteristics but larger differences in selected early app engagement metrics and follow-up weight availability, particularly at weeks 8 and 12 (Additional file 1: Table S4). These findings indicate that missing age data were associated with observed characteristics and follow-up patterns and therefore may not have occurred completely at random. While weight-change prediction and full-retention prediction were largely robust to excluding age, the baseline-only full-retention model showed substantially lower performance without age (ROC AUC, 0.81 vs. 0.64). Its performance and predictor-importance results should therefore be interpreted with caution. Finally, the weight-change prediction analyses were restricted to patients with at least one reported follow-up weight measurement. Patients without any follow-up weight measurement were younger and showed substantially lower recorded app activity during the first four weeks than the included patients. The weight-change prediction models were therefore developed and evaluated in a comparatively more engaged subgroup and may not generalize to patients with limited app engagement. Although the separate full-retention analysis provides complementary information on sustained app use throughout the intervention, it does not eliminate the potential for selection bias in the weight-change prediction analyses.

## Conclusions

In conclusion, this study demonstrates that the predictability of individual outcomes in a real-world digital obesity intervention changes substantially once early-treatment information becomes available. For subsequent weight-loss outcomes, early weight information accounted for most of the improvement in predictive performance beyond baseline characteristics, whereas app engagement provided only limited additional predictive information once 4-week weight was known. In contrast, app engagement substantially improved the prediction of full retention, while early weight information provided no additional benefit. Consistent with these findings, weight-loss response and sustained intervention use were characterized by distinct predictor profiles, suggesting that they represent related but different dimensions of intervention success. These findings provide a basis for further research on the early identification of patients at risk of disengagement, limited weight loss, or both, and for the development of more individualized digital obesity care in which support can be adapted to the predominant risk identified. Importantly, the distinct predictor profiles observed for full retention and clinically meaningful weight loss suggest that practitioners may need to differentiate between strategies aimed at sustaining engagement and those aimed at improving treatment response. Patients at risk of disengagement could, for example, be considered for proactive outreach or additional onboarding support, whereas patients who remain engaged but show limited weight loss may require more intensive or individualized dietary and coaching support.

Before implementation in routine care, however, the developed models require external validation in independent cohorts representing populations and intervention settings relevant to their intended use. Such validation should be conducted in sufficiently large, independent cohorts receiving a comparable digital obesity intervention, with compatible definitions and measurement procedures for the predictors and outcomes required by the original models. The prespecified models should then be applied without refitting, and their performance should be evaluated overall and across relevant patient subgroups. Ideally, validation should subsequently be extended across different time periods and geographic settings to assess the robustness and transportability of model performance. If adequate performance and generalizability are confirmed, prospective impact studies should evaluate whether integrating model predictions into intervention delivery and adapting support accordingly improves patient management, engagement, and weight-loss outcomes compared with usual care. Such studies should also assess whether model-guided care leads to appropriate treatment adaptations and whether these adaptations benefit patients without introducing unintended consequences or inequities.

## Supplementary information

Below is the link to the electronic supplementary material.

Supplementary Material 1

## Data Availability

This study used real-world data from actual therapeutic care. The data sets generated and analyzed during this study are not publicly available because patient consent only allows for the usage of anonymized data by Oviva for research purposes, but precludes making the data publicly available. The underlying code for this study is not publicly available but may be made available to qualified researchers on reasonable request from the corresponding author.

## Abbreviations

BMI: Body mass index
MAE: Mean absolute error
MICE: Multiple imputation by chained equations
ML: Machine learning
RMSE: Root mean squared error
ROC AUC: Area under the receiver operating characteristic curve
SHAP: Shapley additive explanations
SMD: Standardized mean difference
