# Supplementary material for: Machine learning predicts weight change in a real-world digital health application
Source: BMC Med. 2026 Oct 27;24:596. doi: 10.1186/s12916-026-05320-x (PMC13718139; doi:10.1186/s12916-026-05320-x)
Supplement: Supplementary file 1 [file 12916_2026_5320_MOESM1_ESM.docx]

SUPPLEMENTARY INFORMATION

**Machine Learning Predicts** **Weight Change in a Real-World Digital Health Application**

Christina Glasbrenner ^1^, Marco Lehmann ^2^, Christoph Höchsmann ^1^, Felix Schirmann ^2^ and Karsten Koehler ^1^,*

^1^ Technical University of Munich, TUM School of Medicine and Health, Department of Health and Sport Sciences, Munich, Germany

^2^ Oviva AG, Berlin, Germany

*** Correspondence:**

Karsten Koehler, PhD

Technical University of Munich

TUM Campus Olympia Park

Am Olympiacampus 11

80809 Munich

Germany

Phone: +49 89 289 24488

Email: [karsten.koehler@tum.de](mailto:karsten.koehler@tum.de)

**Supplementary Tables**

**Table S1:** Components and delivery characteristics of the Oviva Direkt for Obesity intervention.

| **Intervention component** | **Description** | **Delivery mode** | **Recommended use** | | **Personalization** | | **Corresponding variables in the present study** | |
| --- | --- | --- | --- | --- | --- | --- | --- | --- |
| Professional support | Patients could contact a coach for support with using the application and to discuss individual questions or difficulties. | Individual communication via private in-app chat. | Contact with the coach as needed, for example, when questions or difficulties arose. | | Responses and guidance were tailored to the patient’s individual questions and needs. | | Weekly number of patient messages, coach messages, and patient replies. | |
| Self- monitoring | Patients could log meals, body weight, physical activity, fluid intake, symptoms, mood, and other health-related parameters; graphical summaries displayed changes and progress over time. | Self-administered via the smartphone application. | Recommended frequency varied by parameter (e.g., daily meal logging and weekly body weight logging). | | Recommendations for the frequency of weight, meal, and physical-activity logging could be tailored to the individual patient. | | Body weight at weeks 4, 8, and 12; weekly rec-ommended frequencies for weight, meal, and physical-activity logging. | |
| Self- management | The application supported short-term tasks and goal setting related to diet, physical activity, and other health behaviors. Patients could set and monitor individual goals, including a longer-term target weight. | Self-administered via the smartphone application, with support from app alerts, push notifications, and emails. | Frequency varied by task or goal; some targeted daily behaviors, whereas others involved activities several times per week. | | Intervention-related goals could be individualized and monitored within the application. | | Weekly number of goals set; self-defined target weight at baseline. | |
| Education | Multimedia educational content covered nutrition, physical activity, and behavioral and psychological aspects of weight management. Topics included meal planning and recipes, eating patterns, physical activity, stress management, emotional eating, and sleep. | Text, video, and audio content delivered via the smartphone application. | Thematic educational content was provided weekly, with subsequent content unlocked after completion of the preceding content and associated quiz. | | Meal plans and recipes were personalized to the individual patient. | | Weekly time spent engaging with educational content; weekly recommended number of educational content items. | |
| Feedback | Patients received feedback based on recorded dietary intake, body weight, completion of tasks and goals, and progress over time. Recorded information was also presented in progress displays and weight trajectories, while selected achievements were reinforced through gamification elements. | Primarily automated feedback delivered via the smartphone application. | Feedback was provided either immediately after an activity or based on progress over time. Examples included dietary suggestions following meal logging, reinforcement after achievement of activity goals, and visualization of weight change relative to the individual target weight. | Feedback was informed by self-monitoring data, completed tasks and goals, and progress toward individual targets. | | Weekly number of reactions recorded in the application. | |  |

This overview describes the core intervention components available during the study period from August 2025 to March 2026. The intervention components and delivery characteristics presented here were adapted from the published description of *Oviva Direkt for Obesity* by Lautenbach et al.^[1]^. Recommended use refers to the intended use of the intervention rather than actual app usage. Only app functions corresponding to variables included in the present study are shown in the last column.

**Table S2:** Comparison of baseline characteristics and selected early app engagement metrics between patients included in and excluded from the weight-change prediction analyses.

| Characteristics | Included patients^1^ (n = 17,953) | Excluded patients^2^ (n = 8,761) | Absolute SMD |
| --- | --- | --- | --- |
| Baseline characteristics | | | |
| Age (y) | 53.8 (11.4) | 47.7 (13.0) | 0.496 |
| Sex, n (%)  Female  Male | 14,535 (81)  3,418 (19) | 6,745 (77)  2,016 (23) | 0.098 |
| Height (cm) | 169.0 (8.6) | 169.7 (9.0) | 0.076 |
| Weight (kg) | 100.0 (15.9) | 101.3 (16.8) | 0.074 |
| Body mass index (kg/m^2^) | 34.9 (4.2) | 35.1 (4.5) | 0.033 |
| Self-defined target weight (kg) | 94.0 (15.0) | 95.0 (15.7) | 0.066 |
| Selected app engagement metrics during weeks 1-4^3^ | | | |
| Number of patient messages | 3.3 (5.0) | 0.8 (2.1) | 0.645 |
| Number of coach messages | 14.5 (5.8) | 11.5 (3.8) | 0.612 |
| Number of goals set | 65.8 (45.2) | 14.5 (17.1) | 1.505 |

Continuous variables are presented as mean (SD), and categorical variables as n (%). The magnitude of differences between included and excluded patients was quantified using absolute SMDs.

^1^ Included patients had a recorded baseline weight and at least one recorded follow-up weight measurement at weeks 4, 8, or 12. Patients excluded because of implausible 12-week relative weight-change values based on a 2 × IQR criterion are not shown (n = 71).

^2^ Excluded patients had a recorded baseline weight but no recorded follow-up weight measurement at weeks 4, 8, or 12; that is, all three follow-up measurements were missing.

^3^ Only three selected app engagement metrics are presented. Weekly counts were summed across weeks 1-4.

*Abbreviations:* IQR, interquartile range; SD, standard deviation; SMD, standardized mean difference.

**Table S3:** Number and proportion of patients with missing age data across analysis populations.

| Analysis population | Total patients, n | Patients with missing age data, n (%) |
| --- | --- | --- |
| Full-retention population | 26,714 | 18,628 (69.7) |
| Overall weight-analysis population | 17,953 | 10,268 (57.2) |
| Week 4 weight-analysis sample | 16,701 | 9,370 (56.1) |
| Week 8 weight-analysis sample | 12,829 | 5,956 (46.4) |
| Week 12 weight-analysis sample | 9,806 | 3,433 (35.0) |

**Table S4:** Comparison of baseline characteristics, selected early app engagement metrics, and availability of follow-up weight measurements between patients with and without recorded age in the overall weight-analysis population.

| Characteristics | Patients with recorded age (n = 7,685) | Patients without recorded age (n = 10,268) | Absolute SMD |
| --- | --- | --- | --- |
| Baseline characteristics | | | |
| Sex, n (%)  Female  Male | 6,394 (83)  1,291 (17) | 8,141 (79)  2,127 (21) | 0.100 |
| Height (cm) | 168.7 (8.4) | 169.2 (8.8) | 0.066 |
| Weight (kg) | 100.4 (15.8) | 99.7 (15.9) | 0.044 |
| Body mass index (kg/m^2^) | 35.2 (4.2) | 34.7 (4.2) | 0.115 |
| Self-defined target weight (kg) | 94.3 (14.9) | 93.8 (15.0) | 0.031 |
| Selected app engagement metrics during weeks 1-4^1^ | | | |
| Number of patient messages | 4.3 (5.7) | 2.6 (4.3) | 0.347 |
| Number of coach messages | 15.6 (6.2) | 13.7 (5.3) | 0.326 |
| Number of goals set | 80.2 (47.4) | 55.1 (40.2) | 0.570 |
| Availability of follow-up weight measurements | | | |
| Weight available at week 4, n (%) | 7,331 (95) | 9,370 (91) | 0.166 |
| Weight available at week 8, n (%) | 6,873 (89) | 5,956 (58) | 0.764 |
| Weight available at week 12, n (%) | 6,373 (83) | 3,433 (33) | 1.160 |

Continuous variables are presented as mean (SD), and categorical variables as n (%). The magnitude of differences between patients with and without recorded age was quantified using absolute SMDs.

^1^ Only three selected app engagement metrics are presented. Weekly counts were summed across weeks 1-4.

*Abbreviations:* SD, standard deviation; SMD, standardized mean difference.

**Table S5:** Stability analysis of the top 10 features in the best-performing model for each prediction scenario across cross-validation folds using the full feature set.

| Feature | Rank in final model^1^ | Mean rank ± SD across CV folds | Rank range across CV folds | Top-10 frequency across CV folds |
| --- | --- | --- | --- | --- |
| Clinically meaningful weight loss at week 8 (3% threshold) | | | | |
| BL weight | 1 | 1.0 ± 0.0 | 1-1 | 100% |
| Weight w4 | 2 | 2.0 ± 0.0 | 2-2 | 100% |
| Target weight | 3 | 3.0 ± 0.0 | 3-3 | 100% |
| Multiple weight w2 | 4 | 5.4 ± 1.7 | 4-8 | 100% |
| Number goals w4 | 5 | 5.6 ± 1.1 | 4-7 | 100% |
| Multiple weight w1 | 6 | 9.6 ± 10.4 | 4-28 | 80% |
| Coach messages w1 | 7 | 6.8 ± 1.1 | 5-8 | 100% |
| Multiple weight w4 | 8 | 8.4 ± 2.3 | 6-11 | 80% |
| Patient messages w1 | 9 | 11.6 ± 1.9 | 9-13 | 40% |
| Patient messages w4 | 10 | 12.4 ± 2.8 | 10-17 | 20% |
| Clinically meaningful weight loss at week 8 (5% threshold) | | | | |
| BL weight | 1 | 1.0 ± 0.0 | 1-1 | 100% |
| Weight w4 | 2 | 2.0 ± 0.0 | 2-2 | 100% |
| Target weight | 3 | 3.0 ± 0.0 | 3-3 | 100% |
| Sex | 4 | 6.0 ± 2.2 | 5-10 | 100% |
| Multiple weight w1 | 5 | 12.0 ± 12.9 | 4-34 | 60% |
| Multiple weight w2 | 6 | 11.6 ± 13.2 | 4-35 | 80% |
| Coach messages w1 | 7 | 6.2 ± 1.5 | 4-8 | 100% |
| Multiple weight w4 | 8 | 8.2 ± 1.6 | 7-11 | 80% |
| Height | 9 | 8.8 ± 2.2 | 6-12 | 80% |
| Patient messages w1 | 10 | 9.8 ± 1.5 | 8-12 | 80% |
| Clinically meaningful weight loss at week 12 (3% threshold) | | | | |
| BL weight | 1 | 1.0 ± 0.0 | 1-1 | 100% |
| Weight w4 | 2 | 2.0 ± 0.0 | 2-2 | 100% |
| Target weight | 3 | 3.0 ± 0.0 | 3-3 | 100% |
| Coach messages w1 | 4 | 4.2 ± 0.4 | 4-5 | 100% |
| Patient messages w1 | 5 | 10.0 ± 8.4 | 5-25 | 80% |
| Sex | 6 | 13.0 ± 8.0 | 5-23 | 40% |
| Multiple weight w4 | 7 | 8.0 ± 2.2 | 4-9 | 100% |
| Patient messages w4 | 8 | 10.2 ± 3.4 | 6-13 | 40% |
| Number goals w4 | 9 | 13.6 ± 7.5 | 6-26 | 40% |
| Multiple activity w3 | 10 | 11.2 ± 5.6 | 7-21 | 80% |
| Clinically meaningful weight loss at week 12 (5% threshold) | | | | |
| BL weight | 1 | 1.0 ± 0.0 | 1-1 | 100% |
| Weight w4 | 2 | 2.0 ± 0.0 | 2-2 | 100% |
| Target weight | 3 | 3.0 ± 0.0 | 3-3 | 100% |
| Sex | 4 | 4.2 ± 0.4 | 4-5 | 100% |
| Patient messages w3 | 5 | 6.2 ± 2.3 | 4-10 | 100% |
| Multiple meal w3 | 6 | 9.2 ± 4.9 | 5-17 | 60% |
| Multiple meal w4 | 7 | 10.0 ± 3.4 | 6-15 | 60% |
| Coach messages w1 | 8 | 7.4 ± 1.1 | 6-9 | 100% |
| Multiple meal w1 | 9 | 9.0 ± 3.9 | 5-14 | 60% |
| Patient replies w4 | 10 | 14.8 ± 4.9 | 9-22 | 20% |
| Relative weight change at week 8 | | | | |
| BL weight | 1 | 1.2 ± 0.4 | 1-2 | 100% |
| Weight w4 | 2 | 1.8 ± 0.4 | 1-2 | 100% |
| Coach messages w3 | 3 | 8.2 ± 3.3 | 3-11 | 60% |
| Number goals w4 | 4 | 4.2 ± 1.3 | 3-6 | 100% |
| Multiple weight w1 | 5 | 10.4 ± 7.3 | 5-23 | 80% |
| Target weight | 6 | 5.0 ± 1.4 | 3-7 | 100% |
| Height | 7 | 8.2 ± 1.5 | 6-10 | 100% |
| Number goals w1 | 8 | 4.6 ± 1.9 | 3-8 | 100% |
| Learn time w4 | 9 | 8.6 ± 2.7 | 6-12 | 60% |
| Age | 10 | 9.0 ± 3.3 | 4-13 | 80% |
| Relative weight change at week 12 | | | | |
| BL weight | 1 | 1.0 ± 0.0 | 1-1 | 100% |
| Weight w4 | 2 | 2.0 ± 0.0 | 2-2 | 100% |
| Target weight | 3 | 3.0 ± 0.0 | 3-3 | 100% |
| Sex | 4 | 4.2 ± 0.4 | 4-5 | 100% |
| Coach messages w1 | 5 | 4.8 ± 0.4 | 4-5 | 100% |
| Patient messages w1 | 6 | 9.8 ± 4.8 | 6-18 | 80% |
| Multiple weight w4 | 7 | 9.8 ± 4.3 | 6-15 | 60% |
| Multiple learn w3 | 8 | 8.8 ± 2.4 | 6-12 | 80% |
| Multiple learn w4 | 9 | 9.6 ± 3.0 | 6-13 | 60% |
| Number goals w4 | 10 | 12.2 ± 5.2 | 8-20 | 60% |
| Full retention | | | | |
| Number goals w4 | 1 | 1.0 ± 0.0 | 1-1 | 100% |
| Age | 2 | 2.0 ± 0.0 | 2-2 | 100% |
| Multiple meal w4 | 3 | 3.0 ± 0.0 | 3-3 | 100% |
| Learn time w4 | 4 | 4.0 ± 0.0 | 4-4 | 100% |
| Multiple meal w3 | 5 | 6.8 ± 1.3 | 6-9 | 100% |
| Number goals w3 | 6 | 5.6 ± 0.9 | 5-7 | 100% |
| Learn time w2 | 7 | 6.8 ± 1.3 | 5-8 | 100% |
| Learn time w3 | 8 | 8.6 ± 2.5 | 5-11 | 80% |
| Learn time w1 | 9 | 8.6 ± 0.5 | 8-9 | 100% |
| Multiple weight w4 | 10 | 10.6 ± 2.2 | 7-13 | 20% |

^1^ The final model was refitted on the full training set after cross-validation and therefore does not correspond to an individual cross-validation fold.

*Abbreviations:* BL, baseline; CV, cross-validation; SD, standard deviation; w1, week 1; w2, week 2; w3, week 3; w4, week 4.

**Table S6:** Predictive performance across feature sets for subsequent attainment of clinically meaningful weight-loss thresholds.

| Prediction outcome | BL | BL + 4-week weight | | BL + app engagement^1^ | | BL + 4-week weight + app engagement^1^ | |
| --- | --- | --- | --- | --- | --- | --- | --- |
| ≥3% weight loss at week 8 among participants with <3% weight loss at week 4^2^ | 0.55 | | 0.86 | | 0.61 | | 0.86 |
| ≥5% weight loss at week 8 among participants with <5% weight loss at week 4^2^ | 0.57 | | 0.94 | | 0.65 | | 0.94 |
| ≥3% weight loss at week 12 among participants with <3% weight loss at week 4^2^ | 0.55 | | 0.83 | | 0.63 | | 0.83 |
| ≥5% weight loss at week 12 among participants with <5% weight loss at week 4^2^ | 0.55 | | 0.88 | | 0.64 | | 0.88 |

^1^ Weekly app engagement metrics recorded during the first four weeks of the intervention were included.

^2^ Weight-loss thresholds refer to relative weight change from baseline. Only the ROC AUC of the best-performing model is shown for each feature set. Higher ROC AUC values indicate better classification performance.

*Abbreviations:* BL, baseline; ROC AUC, area under the receiver operating characteristic curve.

**Table S7:** Sensitivity analysis of binary classification performance for weight change prediction after excluding age as a predictor, using baseline-only and full-feature predictor sets.

|  |  | ROC AUC | |  |  | |
| --- | --- | --- | --- | --- | --- | --- |
| Prediction outcome | **Predictor set** | **Primary analysis (including age)^1^** | **Sensitivity analysis (excluding age)^2^** | | | **Δ^3^** |
| Week 4 (3% threshold) | BL | 0.55 | 0.55 | | | 0.00 |
| Week 8 (3% threshold) | BL | 0.54 | 0.51 | | | 0.03 |
| Week 8 (3% threshold) | Full feature set^4^ | 0.88 | 0.89 | | | -0.01 |
| Week 8 (5% threshold) | BL | 0.58 | 0.54 | | | 0.04 |
| Week 8 (5% threshold) | Full feature set^4^ | 0.92 | 0.91 | | | 0.01 |
| Week 12 (3% threshold) | BL | 0.57 | 0.56 | | | 0.01 |
| Week 12 (3% threshold) | Full feature set^4^ | 0.87 | 0.87 | | | 0.00 |
| Week 12 (5% threshold) | BL | 0.54 | 0.54 | | | 0.00 |
| Week 12 (5% threshold) | Full feature set^4^ | 0.88 | 0.87 | | | 0.01 |

^1^ Only the best-performing model from the primary analysis is shown for each prediction scenario and predictor set.

^2^ For the sensitivity analysis, the best-performing model identified in the primary analysis for each prediction scenario and predictor set was refitted after excluding age as a predictor.

^3^ Δ ROC AUC was calculated as ROC AUC from the primary analysis minus ROC AUC from the sensitivity analysis excluding age.

^4^ The full feature set included baseline variables, 4-week weight, and app engagement data recorded during the first four weeks of the intervention.

*Abbreviations:* BL, baseline; ROC AUC, area under the receiver operating characteristic curve.

**Table S8:** Predictive performance across feature sets for subsequent relative weight change.

| Prediction outcome | BL | BL + 4-week weight | | BL + app engagement^1^ | | BL + 4-week weight + app engagement^1^ | |
| --- | --- | --- | --- | --- | --- | --- | --- |
| Subsequent relative weight change from week 4 to week 8^2^ | 0.0150 | | 0.0143 | | 0.0147 | | 0.0142 |
| Subsequent relative weight change from week 4 to week 12^2^ | 0.0226 | | 0.0210 | | 0.0227 | | 0.0212 |

^1^ Weekly app engagement metrics recorded during the first four weeks of the intervention were included.

^2^ Only the RMSE of the best-performing model is shown for each feature set. Lower RMSE values indicate better regression performance.

*Abbreviations:* BL, baseline; RMSE, root mean squared error.

**Table S9:** Sensitivity analysis of regression performance for relative weight change prediction after excluding age as a predictor, using baseline-only and full-feature predictor sets.

|  |  | RMSE | |  | |  |
| --- | --- | --- | --- | --- | --- | --- |
| Prediction outcome | **Predictor set** | **Primary analysis (including age)^1^** | **Sensitivity analysis (excluding age)^2^** | | **Δ^3^** | |
| Relative weight change at week 4 | BL | 0.018 | 0.018 | | 0.000 | |
| Relative weight change at week 8 | BL | 0.029 | 0.029 | | 0.000 | |
| Relative weight change at week 8 | Full feature set^4^ | 0.022 | 0.023 | | -0.001 | |
| Relative weight change at week 12 | BL | 0.032 | 0.032 | | 0.000 | |
| Relative weight change at week 12 | Full feature set^4^ | 0.022 | 0.022 | | 0.000 | |

^1^ Only the best-performing model from the primary analysis is shown for each prediction scenario and predictor set.

^2^ For the sensitivity analysis, the best-performing model identified in the primary analysis for each prediction scenario and predictor set was refitted after excluding age as a predictor.

^3^ Δ RMSE was calculated as RMSE from the primary analysis minus RMSE from the sensitivity analysis excluding age.

^4^ The full feature set included baseline variables, 4-week weight, and app engagement data recorded during the first four weeks of the intervention.

*Abbreviations:* BL, baseline; RMSE, root mean squared error.

**Table S10:** Sensitivity analysis of binary classification performance for full retention prediction after excluding age as a predictor, using baseline-only and full-feature predictor sets.

|  |  | ROC AUC | |  |  | |
| --- | --- | --- | --- | --- | --- | --- |
| Prediction outcome | **Predictor set** | **Primary analysis (including age)^1^** | **Sensitivity analysis (excluding age)^2^** | | | **Δ^3^** |
| Full retention | BL | 0.81 | 0.64 | | | 0.17 |
| Full retention | Full feature set^4^ | 0.90 | 0.89 | | | 0.01 |

^1^ Only the best-performing model from the primary analysis is shown for each prediction scenario and predictor set.

^2^ For the sensitivity analysis, the best-performing model identified in the primary analysis for each prediction scenario and predictor set was refitted after excluding age as a predictor.

^3^ Δ ROC AUC was calculated as ROC AUC from the primary analysis minus ROC AUC from the sensitivity analysis excluding age.

^4^ The full feature set included baseline variables, 4-week weight, and app engagement data recorded during the first four weeks of the intervention.

*Abbreviations:* BL, baseline; ROC AUC, area under the receiver operating characteristic curve.

**Supplementary Figures**

**Fig. S1:** Distribution of patients in the weight-analysis population across weight loss categories defined by 3%, 5%, and 10% thresholds at 4, 8, and 12 weeks.

**Fig. S2:** Distribution of relative weight change in the weight-analysis population at 4, 8, and 12 weeks compared with the baseline.

*Note:* The figure shows relative weight changes truncated to the range −15% to +15% for visualization purposes. Values outside this range are not displayed (affecting only extreme outliers).

A

|  |  | **4 weeks** | | | |  | **8 weeks** | | | |  | **12 weeks** | | | |
| --- | --- | --- | --- | --- | --- | --- | --- | --- | --- | --- | --- | --- | --- | --- | --- |
|  |  | LR | DT | RF | GB |  | LR | DT | RF | GB |  | LR | DT | RF | GB |
| $\boldsymbol{3\%}$ | ROC AUC  Accuracy  Sensitivity  Specificity |  | | | |  |  | | | |  |  | | | |
| $\boldsymbol{5\%}$ | ROC AUC  Accuracy  Sensitivity  Specificity | n/a | | | |  |  | | | |  |  | | | |

B

|  |  | **4 weeks** | | | |  | **8 weeks** | | | |  | **12 weeks** | | | |
| --- | --- | --- | --- | --- | --- | --- | --- | --- | --- | --- | --- | --- | --- | --- | --- |
|  |  | LR | DT | RF | GB |  | LR | DT | RF | GB |  | LR | DT | RF | GB |
| $\boldsymbol{3\%}$ | ROC AUC  Accuracy  Sensitivity  Specificity | n/a | | | |  |  | | | |  |  | | | |
| $\boldsymbol{5\%}$ | ROC AUC  Accuracy  Sensitivity  Specificity | n/a | | | |  |  | | | |  |  | | | |

**Fig. S3:** Heatmap summarizing binary classification performance metrics for predicting weight change across weight-loss thresholds of 3% (at 4, 8, and 12 weeks) and 5% (at 8 and 12 weeks). Panel A shows performance metrics using baseline variables only, whereas panel B shows performance metrics using baseline variables, 4-week weight, and app engagement data recorded during the first four weeks of the intervention.

*Abbreviations:* LR, logistic regression; DT, decision tree; RF, random forest; GB, gradient boosting; ROC AUC, area under the receiver operating characteristic curve; n/a, not available.

A	B

C	D

E	F

G	H

**Fig. S4:** SHAP beeswarm plots for the best-performing full-feature model in each scenario predicting clinically meaningful weight loss (≥3% and ≥5%). Full-feature models included baseline variables, 4-week weight, and app engagement metrics recorded during the first four weeks of the intervention. Panels A-B show SHAP summary plots for 8-week prediction using the 3% threshold, with panel A displaying the 10 most important predictors ranked by model-derived feature importance and panel B providing a focused view of mid-ranked predictors (ranks 3–10). Panels C-D show corresponding results for the 5% threshold at 8 weeks. Panels E-F and G-H present analogous SHAP summaries for 12-week predictions using 3% and 5% thresholds, respectively. Positive SHAP values indicate an increase in the predicted probability of achieving the respective weight-loss outcome (≥3% or ≥5% weight loss), whereas negative values indicate a decrease in predicted probability.

*Abbreviations:* BL, baseline; SHAP, SHapley Additive exPlanations; w1, week 1; w2, week 2; w3, week 3; w4, week 4.

A

|  | **4 weeks** | | |  | **8 weeks** | | |  | **12 weeks** | | |
| --- | --- | --- | --- | --- | --- | --- | --- | --- | --- | --- | --- |
|  | Bias | MAE | RMSE |  | Bias | MAE | RMSE |  | Bias | MAE | RMSE |
| LR  Ridge  Lasso  ElasticNet  DT  RF  GB |  | | |  |  | | |  |  | | |

B

|  | **4 weeks** | | |  | **8 weeks** | | |  | **12 weeks** | | |
| --- | --- | --- | --- | --- | --- | --- | --- | --- | --- | --- | --- |
|  | Bias | MAE | RMSE |  | Bias | MAE | RMSE |  | Bias | MAE | RMSE |
| LR  Ridge  Lasso  ElasticNet  DT  RF  GB | n/a | | |  |  | | |  |  | | |

**Fig. S5:** Heatmap summarizing regression performance metrics for predicting relative weight change at weeks 4, 8, and 12. Panel A shows performance using baseline variables only, whereas panel B shows performance using the full predictor set, comprising baseline variables, 4-week weight, and app engagement data recorded during the first four weeks of the intervention. For week-4 prediction, only baseline variables were used because early intervention data were not yet available.

*Abbreviations:* LR, linear regression; ElasticNet, elastic net-regularized linear regression; DT, decision tree; RF, random forest; GB, gradient boosting; MAE, mean absolute error; RMSE, root mean squared error; n/a, not available.

1. Lautenbach A, Blüher M, Mijuskovic A, Jones L, Schirmann F. Efficacy of a digital health application for weight management in people with obesity: 6-months results from a randomized controlled trial. Int J Obes. 2026;50:466–73. https://doi.org/10.1038/s41366-025-01967-3.
